# The Adipocytokine Nampt and Its Product NMN Have No Effect on Beta-Cell Survival but Potentiate Glucose Stimulated Insulin Secretion

**DOI:** 10.1371/journal.pone.0054106

**Published:** 2013-01-16

**Authors:** Robert Spinnler, Theresa Gorski, Katharina Stolz, Susanne Schuster, Antje Garten, Annette G. Beck-Sickinger, Marten A. Engelse, Eelco J. P. de Koning, Antje Körner, Wieland Kiess, Kathrin Maedler

**Affiliations:** 1 Center for Pediatric Research Leipzig (CPL), Department for Women and Child Health, University of Leipzig, Leipzig, Germany; 2 Leipzig University Medical Center, IFB Adiposity Diseases, Leipzig, Germany; 3 Center for Biomolecular Interactions, University of Bremen, Bremen, Germany; 4 Institute of Biochemistry, Faculty of Bioscience, Pharmacy and Psychology, University of Leipzig, Leipzig, Germany; 5 Department of Nephrology, Leiden University Medical Center, Leiden, The Netherlands; 6 Hubrecht Institute, Utrecht, The Netherlands; Tohoku University, Japan

## Abstract

**Aims/Hypothesis:**

Obesity is associated with a dysregulation of beta-cell and adipocyte function. The molecular interactions between adipose tissue and beta-cells are not yet fully elucidated. We investigated, whether or not the adipocytokine Nicotinamide phosphoribosyltransferase (Nampt) and its enzymatic product Nicotinamide mononucleotide (NMN), which has been associated with obesity and type 2 diabetes mellitus (T2DM) directly influence beta-cell survival and function.

**Methods:**

The effect of Nampt and NMN on viability of INS-1E cells was assessed by WST-1 assay. Apoptosis was measured by Annexin V/PI and TUNEL assay. Activation of apoptosis signaling pathways was evaluated. Adenylate kinase release was determined to assess cytotoxicity. Chronic and acute effects of the adipocytokine Nampt and its enzymatic product NMN on insulin secretion were assessed by glucose stimulated insulin secretion in human islets.

**Results:**

While stimulation of beta-cells with the cytokines IL-1β, TNFα and IFN-γ or palmitate significantly decreased viability, Nampt and NMN showed no direct effect on viability in INS-1E cells or in human islets, neither alone nor in the presence of pro-diabetic conditions (elevated glucose concentrations and palmitate or cytokines). At chronic conditions over 3 days of culture, Nampt and its product NMN had no effects on insulin secretion. In contrast, both Nampt and NMN potentiated glucose stimulated insulin secretion acutely during 1 h incubation of human islets.

**Conclusion/Interpretation:**

Nampt and NMN neither influenced beta-cell viability nor apoptosis but acutely potentiated glucose stimulated insulin secretion.

## Introduction

Obesity and the development of type 2 diabetes mellitus (T2DM) are strongly related. It has been suggested, that molecular signals from adipose tissue convey the information that beta-cells reside in an obese environment. T2DM results from a pancreatic islet failure to produce sufficient amounts of insulin and from a decrease in the sensitivity of glucose-metabolizing tissues to insulin [1]. A failure of beta-cell function and a reduction in beta-cell mass mainly caused by apoptosis are two of the factors underlying the complex etiology of T2DM. They are often associated with an increase in circulating cytokines, free fatty acids (FFAs) and chronic hyperglycaemia [2]. Obesity leads to dysregulation of adipose tissue function, up regulation of proinflammatory cytokine release and enhanced secretion of FFAs which all might contribute to pancreatic beta-cell damage.

Cytokines, alone or in combination, take part in the pathogenesis of diabetes causing pancreatic beta-cell dysfunction and decline of viability [3]–[5].

Additionally, gluco-lipotoxicity causes beta-cell failure in T2DM [6], and also saturated FFAs alone cause beta-cell apoptosis [7]–[10], whereas the monounsaturated FFA oleate is less toxic [7], [10] and even protects against palmitate-induced apoptosis in beta-cells [10].

A metabolic dysregulation also results in an altered production and secretion of adipocytokines, which *per se* influences beta-cell survival and function. Specifically, the adipocytokines leptin and adiponectin influence beta-cell survival and death [9], [11], [12]. Leptin, secreted from white adipocytes, is an essential factor in regulating body weight and glucose homeostasis [12]. Leptin receptors are expressed by beta-cells [13]. *In vitro*, leptin stimulates the release of IL-1β, decreases the expression of the IL-1 receptor antagonist (IL-1Ra) in human islets [14] and upon chronic exposure induces beta-cell apoptosis [11] and impairs islet function in rodent and human beta-cells [9], [11], [12]. In INS-1 cells leptin alone does not modify caspase-3 activation or DNA fragmentation, whereas a combination of leptin with cytokines or FFAs suppress cytokine and palmitate induced apoptosis and DNA fragmentation [9]. While leptin levels are elevated in obesity, adiponectin is decreased [15] and correlates with impaired beta-cell function and survival. It binds to two subtypes of adiponectin receptors, AdipoR1 and AdipoR2. AdipoR1 is expressed in muscle, while AdipoR2 is mainly expressed in the liver and in beta-cells at similar levels [16]. *In vivo*, adiponectin exists as a globular fragment or as full-length form [17]. The C-terminal globular domain of adiponectin, gAcrp30, counteracts cytokine- and palmitate-induced beta-cell apoptosis [9], increases insulin secretion in islets from high fat diet-treated mice at high glucose concentrations [18], [19] and prevents cytokine- and FFA-induced suppression of insulin secretion in INS-1 cells [9]. In contrast, adiponectin administration to human islets fails to prevent beta-cell apoptosis induced by FFAs [20].

Nicotinamide phosphoribosyltransferase (Nampt, also known as PBEF or visfatin) has been identified as a novel adipokine, with both intra- and extracellular enzymatic function. Rather than exerting insulin-mimetic effects *in vitro* or *in vivo,* Nampt catalyses the rate-limiting step in mammalian NAD biosynthesis [21]. Nicotinamide mononucleotide (NMN), the enzyme product of Nampt, has been shown to correct impaired islet function in Nampt^(+/−)^ mice [21] and to restore suppressed insulin secretion in mouse models of impaired beta-cell function [22]. Nampt also acts in a cytokine-like manner, either in an anti-apoptotic [23], [24] or a pro-inflammatory fashion [25]. Nampt levels in the circulation are elevated in non-obese and obese T2DM patients and correlate with increased IL-6 serum levels [26].

Since previous studies yielded contradicting data, we aimed to evaluate whether or not the adipocytokines leptin, adiponectin, Nampt or its enzyme product NMN affect beta-cell viability, cytotoxicity, apoptosis and beta-cell function.

## Materials and Methods

### Ethics Statement

We have received the human islets from the European Consortium for Islet Transplantation (ECIT), where six European-based centers for human islet transplantation have established collaboration with the aim establishing an integrated project to develop and expand clinical islet transplantation. Whenever islet isolation fails to be suitable for transplantation, centers provide them for islet research. Thus, these research projects apply to NIH regulations PHS 398, exemption 4. Human pancreata are harvested from brain dead donors, according to the European and National regulations for organ procurement. Human islet isolations are performed through approved protocols of the centers. Donors or their family members have given written consent to donate organs for transplantation and research, all documented by the transplantation centers. For this study, all islet preparations were received from the University of Leiden. The University of Bremen institutional review board specifically approved this study.

### Cell Culture

The rat insulinoma cell line INS-1E was a generous gift from Prof. Claes Wollheim, Geneva, Switzerland and represents a highly differentiated clone of INS-1 cells [27]. INS-1E cells (passages 70–95) were grown in RPMI 1640 (PAA Laboratories, Pasching, Austria) culture medium containing 25 mM HEPES and supplemented with 2 mM L-glutamine (PAA), 50 µM β-mercaptoethanol (Sigma, Munich, Germany), 1 mM sodium pyruvate (Sigma) and 5% FCS (PAA) in an atmosphere of 5% CO_2_ at 37°C. Subconfluent cells were maintained in culture by passaging of cultures every 3–4 days after plating.

Human islets were isolated from pancreata of four non-diabetic organ donors at the Leiden University Medical Center and cultured as described previously [28]. For long-term *in vitro* studies, islets were cultured on extracellular matrix–coated plates derived from bovine corneal endothelial cells (Novamed Ltd., Jerusalem, Israel), allowing the cells to attach to the dishes and spread, preserving their functional integrity.

Cells were cultured with IL-1β [0.1–100 ng/ml], IFN-γ [0.1–100 ng/ml] and TNFα [0.1–100 ng/ml] (R&D Systems, McKinley Place, MN, USA) or the adipocytokines leptin [1–500 ng/ml], gAcrp30 [1.67–835 ng/ml] (both PeproTech GmbH, Hamburg, Germany) or Nampt [1–25 ng/ml] (kindly provided by AdipoGen Inc., Incheon, South Korea) or the enzymatic product NMN [10–1000 µM] (Sigma) or camptothecin [2 µM] (Sigma) and etoposide [85 µM] (Calbiochem, Merck KGaA, Darmstadt, Germany) or palmitate [0.125–1 mM] and oleate [0.125–1 mM] (Sigma), dissolved as described previously [28].

### Cell Viability, Cytotoxicity and Apoptosis

To measure viability and cytotoxicity, cells were seeded into 96well plates at 25,000 cells/well for 72 hours. Cells were incubated for 24 h in RPMI 1640 medium without FCS, but supplemented with 0.2% BSA (Life Technologies GmbH, Darmstadt, Germany) and then incubated in serum free medium in the absence or presence of the treatment conditions for 48–72 h. Viability and cytotoxicity were measured by WST-1 assay (Roche, Mannheim, Germany) and by ToxiLight®BioAssay Sample Kit (Lonza Inc., Rockford, IL,USA) respectively, according to manufacturer’s instructions. Apoptosis in INS-1E cells was assessed by FITC-AnnexinV (An) and propidium iodide (PI) staining (BD, Heidelberg, Germany) and flow cytometric analysis (BD FACSCalibur). For each sample, 10,000 cells were counted. An-positive and double-stained An/PI positive cells were considered to be apoptotic. For detection of beta-cell apoptosis in human islets, 100 human islets were cultured in suspension dishes, treated for 72 h, and fixed with Bouińs solution. Islet sections were prepared as described previously [29] and insulin and TUNEL staining was performed according to the manufacturer (In Situ Cell Death Detection Kit, TMR-red; Roche) [29].

### Measurement of Intracellular NAD Level

The concentrations of NAD in the whole cell extracts were analysed by a commercially available NAD/NADH assay kit (EnzyChrom™ NAD/NADH Assay Kit; Köln, Germany). Therefore, 500,000 cells/well were seeded in 6well plates and cultured as described above. After treatment for 2 or 48 h, cells were trypsinized and 2 wells per sample were pooled and lysed in 100 µl NAD extraction buffer. To homogenize the samples, cell extracts were undergo freeze/thaw cycles. NAD level were determined according to manufacturer’s instructions. The cell pellet of each sample was resuspended in 100 µl 2% SDS, shaked for 10 min at 99°C and centrifuged for 5 min at 20,000×g and then used for protein determination (BCA Assay, Pierce Thermo Scientific). The NAD level of each sample was referred to the corresponding total protein amount of the sample.

### Western Blotting

INS-1E cells were seeded into 6well plates at 500,000 cells/w ell and grown in culture medium. After 72 h, cells were incubated in serum free medium for 24 h. Thereafter, cells were incubated for 48 h for activated caspase-3, 6 h for p53 and 3 h for NF-κB detection under serum free conditions in the absence or presence of the treatment conditions. Equal amounts of protein from each treatment group were run on 10% or 15% SDS–polyacrylamide gels. After semi-dry transfer onto nitrocellulose, membranes (0.45 µm) were blocked and subsequently incubated with rabbit anti-phospho-p53 antibody (Ser15), rabbit anti-caspase-3 antibody, rabbit anti-phospho-NF-κB-p65 (Ser536) antibody, rabbit anti-NF-κB-p65 antibody (all Cell Signaling Technology Inc., Beverly, MA, USA), mouse anti-β-actin antibody (Sigma) or mouse anti-GAPDH antibody (Millipore, Billerica, USA) over night, followed by a 2 h incubation with anti-rabbit or anti-mouse IgG HRP-conjugated antibodies (Dako A/S, Glostrup, Denmark). Specific bands were visualized using ECL chemiluminescence substrate (Super Signal Pico, Pierce, USA) and CL-XPosure film (Thermo Scientific, Waltham, MA, USA).

### Insulin Secretion Assays

Human islets used to perform glucose and IBMX/Forskolin stimulated insulin secretion (GSIS) experiments were kept in culture medium on matrix-coated plates derived from bovine corneal endothelial cells (Novamed Ltd.). For determining the chronic effects of the adipocytokines, islets were exposed for 72 h and then washed and pre-incubated (30 min) in Krebs Ringer bicarbonate buffer (KRB) containing 2.8 mM glucose and 0.5% BSA. For acute insulin release in response to glucose, islets were washed, KRB was then replaced by KRB 2.8 mM glucose for 1 h (basal), followed by an additional 1 h-incubation in KRB 16.7 mM glucose (stimulated).

To assess the acute effects of the adipocytokines, human islets were incubated after a 2 day- pre-incubation and recovery period for 1 h at 2.8 mM glucose, followed by a 1 h-incubation period at 2.8 mM glucose plus adipocytokines and an additional 1 h-incubation period at 16.7 mM glucose plus adipocytokines.

A second parallel experiment was designed to directly compare the adipocytokine effects after glucose stimulation. Human islets were acutely incubated with 2.8 mM for 1 h, followed by incubation at 16.7 mM glucose for 1 h and incubation at 16.7 mM glucose plus adipocytokines for 1 h.

A third parallel experiment was designed to investigate whether adipokines also influence secretory machinery in general. Human islets were acutely incubated with 2.8 mM for 1 h, followed by incubation at in the presence of IBMX (100 uM) and Forskolin (10 uM, Sigma) as described before [30].

Islets were extracted with 0.18 N HCl in 70% ethanol for determination of insulin content. Islet insulin was determined using human insulin ELISA (ALPCO, Salem, NH, USA) and expressed per content.

### Statistical Analyses

Significant differences were determined using GraphPad Prism software 4 and the unpaired Student’s t-test or by one-way ANOVA with Bonferroni’s Multiple Comparison Test as posthoc test. The threshold of significance was set p<0.05.

## Results

### The Adipocytokines Leptin, Adiponectin, Nampt and NMN have no Direct Effects on Beta-cell Survival in INS-1E Cells

First, we confirmed the presence of the adiponectin receptors AdipoR1 and AdipoR2 as well as the leptin receptor (OB-R, LeptinR) in INS-1E cells (Fig. S1) [14], [20], whereas the existence of a specific receptor for Nampt is currently unknown.

**Figure 1:**
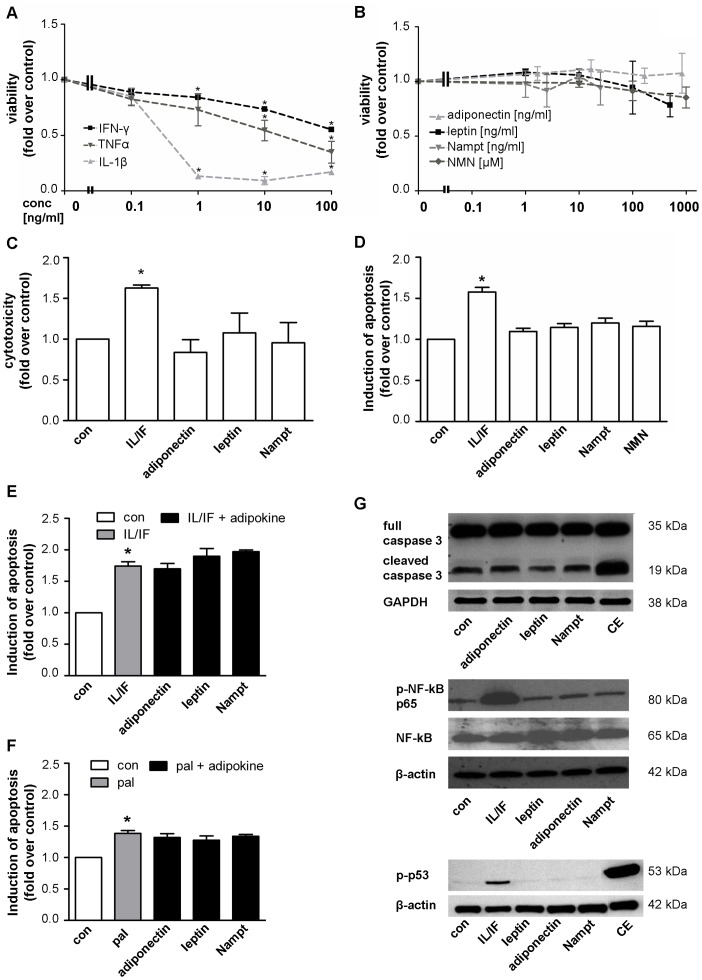
The adipocytokines leptin, adiponectin, Nampt and NMN have no direct effects on beta-cell survival in INS-1E cells. INS-1E cells were kept under serum-free conditions 24 h before and during the 48 h experiment. (**A,B**) INS-1E cells were exposed to cytokines (**A**: IL-1β, IFN-γ or TNFα) or adipocytokines (**B**: adiponectin, leptin, Nampt, NMN) at the indicated concentrations for 48 h and cell viability was measured by WST-1 assay. Data are shown as means ±SEM of 3 independent experiments performed in triplicates. Statistical analyses were performed by one-way ANOVA with Bonferroni’s Multiple Comparison Test as posthoc test. **C,D**: INS-1E cells were exposed to adipocytokines (adiponectin 167 ng/ml, leptin 200 ng/ml, Nampt 2.5 ng/ml, NMN 100 µM) or a cytokine combination (10 ng/ml IL-1β+10 ng/ml IFN-γ) for 48 h. Cytotoxicity (**C**) was analyzed by measuring the release of adenylate kinase into the supernatant and (**D**) apoptosis was measured by FITC-annexinV (An) and propidium iodide (PI) staining and subsequent flow cytometric analysis of An-positive and double An/PI positive cells. Results were expressed relative to cells exposed to serum free medium (con) and as means ±SEM of three independent experiments performed in triplicates. **E,F**: INS-1E cells were exposed to a cytokine combination (IL/IF; 10 ng/ml IL-1β+10 ng/ml IFN-γ) (**E**) or 0.25 mM palmitate (pal) (**F**) for 48 h in the absence or presence of the adipocytokines (167 ng/ml adiponectin, 200 ng/ml leptin, 2.5 ng/ml Nampt) and induction of apoptosis was measured by An/PI staining and flow cytometric analysis. Data are shown as means ±SEM of triplicates of three independent experiments. Statistical analyses were performed by student´s t-test. G: INS-1E cells were exposed to the adipocytokines adiponectin (167 ng/ml), leptin (200 ng/ml) or Nampt (2.5 ng/ml) or a combination of camptothecin (2 µM) and etoposide (85 µM; CE, **upper and lower panel**) or a cytokine combination (10 ng/ml IL-1β+10 ng/ml IFN-γ, **middle and lower panel**). Western blot analyses were performed for full length and cleaved caspase-3 (**upper panel**), phospho-NF-κB p65 (Ser536) and NF-κB p65 (**middle panel**) and phospho-p53 (Ser15) (**lower panel**). GAPDH or beta-actin were used as loading control. All panels show one typical blot out of three independent experiments. *p<0.05 compared to untreated control.

Cell viability in INS-1E cells was reduced by the cytokines IL-1β, IFN-γ and TNFα during 48 h exposure in a dose-dependent manner. IL-1β and IFN-γ reduced beta-cell viability starting at a concentration of 1 ng/ml and TNFα at a higher concentration of 10 ng/ml. At a cytokine concentration of 10 ng/ml the viability of INS-1E cells was reduced by 91.4±1.7% by IL-1β stimulation, 45.6±6.3% by TNFα and 26.3±2.0% by IFN-γ, respectively (Fig. 1A). For further experiments, a cytokine combination of IL-1β (10 ng/ml) and IFN-γ (10 ng/ml) was used as control. In contrast, the adipocytokines leptin, adiponectin, Nampt and NMN showed no effect on viability over a wide range of concentrations (Fig. 1B) at 48 h long-term exposure.

These results were confirmed by analyzing cytotoxicity and apoptosis during the treatment. Cytotoxicity was investigated by measuring the release of adenylate kinase from damaged cells and apoptosis by An/PI labeling and subsequent flow cytometric analysis. Analyses of cytotoxicity and apoptosis confirmed the toxic effects of the cytokines but not of adipocytokines on beta-cell survival (Fig. 1C,D, Fig. S3). Concentrations of the adipocytokines were chosen according to physiological levels (see Discussion). The cytokine combination IL-1β and IFN-γ (10 ng/ml each) induced cytotoxic (60.9±10.5%, Fig. 1C) as well as apoptotic (63.0±9.0%, Fig. 1D) effects after 48 h stimulation. To investigate whether adipocytokines may protect INS-1E cells from cytokine- or palmitate-induced apoptosis or may enhance apoptosis, INS-1E cells were stimulated with a combination of adipocytokines and cytokines or palmitate for 48 h. The apoptosis induced by cytokines (IL-1β/IFN-γ) and palmitate could not be ameliorated by the adipocytokines leptin, adiponectin or Nampt (Fig. 1E,F). Western blotting analysis revealed similar lacking effects of the adipocytokines adiponectin, leptin, or Nampt on different apoptotic pathways (Fig. 1G). The combination of cytokines IL-1β/IFN-γ or the pro-apoptotic cocktail of camptothecin and etoposide were used as positive controls for activation of different apoptotic pathways, such as cleavage of caspase-3 (Fig. 1G, upper panel), phosphorylation of NF-κB p65 subunit (Ser536) (Fig. 1G, middle panel) and of p53 (Ser15) (Fig. 1G, lower panel). To evaluate whether the INS-1E cell model used in our study is able to activate mechanisms to counteract apoptosis, we also investigated the protective effects of oleate on palmitate-induced cytotoxicity (Fig. S2A–C). A previously observed protection of beta-cells from palmitate-induced apoptosis by oleate [8], [11], [31] could have been confirmed in our study in INS-1E cells (Fig. S2D).

### Nampt and NMN have no Direct Effects on Beta-cell Survival in Human Islets

**Figure 2:**
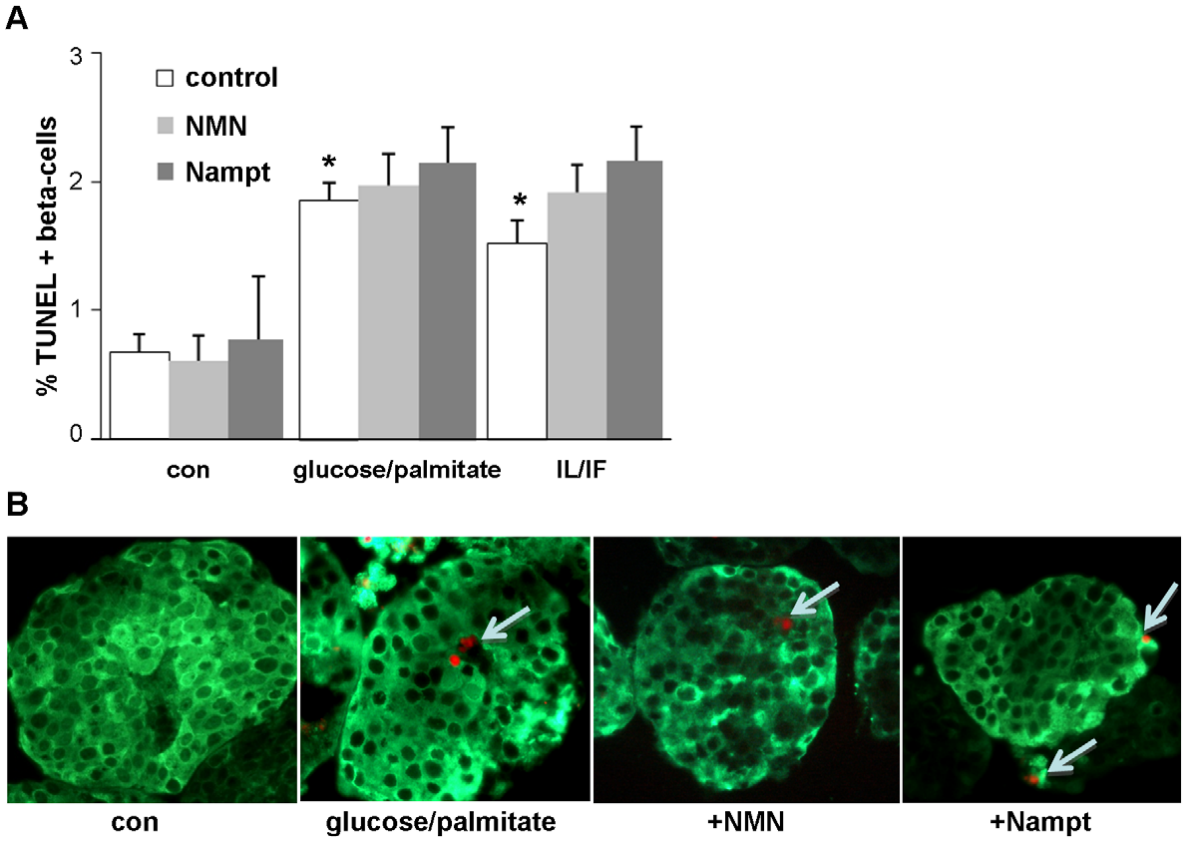
Nampt and NMN have no direct effects on beta-cell survival in human islets. (**A,B**) Human pancreatic islets were cultured in suspension with the mixture of 22.2 mM glucose/0.5 mM palmitate or 2 ng/ml IL-1β/1000 IU IFN-γ in the absence (con) or presence of NMN (100 µM) or Nampt (2.5 ng/ml) for 72 h. Apoptosis was analysed in paraffin embedded islet sections by the TUNEL assay (red nuclei, white arrows) and counter-stained in green for insulin (**B**). Results are means ±SEM of the %TUNEL-positive beta-cells (the average number of beta-cells counted were 1456±277 for each treatment group in each experiment) of three different experiments from three different organ donors. **B:** shows representative staining pictures. *p<0.05 compared to vehicle treated control.

Survival data from cell lines are difficult to extrapolate to primary cells. While Nampt and NMN did not induce beta-cell apoptosis in INS-1E cells, the direct effects of Nampt and NMN on the human beta-cell are unknown. Our next experiments investigated the effects of Nampt and NMN under control and diabetogenic conditions on beta-cell survival in human islets. The same physiological concentrations were used as in the cell line experiments. In addition to Nampt, we also exposed human islets to its enzymatic product NMN for 72 h. In confirmation with our results obtained in INS-1E cells, no effect of Nampt or NMN was observed on beta-cell apoptosis in human islets at control conditions (5.5 mM glucose) (Fig. 2A). Apoptosis was induced by 72 h exposure of human islets to the mixture of 22.2 mM glucose and 0.5 mM palmitate which induced a 2.7-fold induction in beta-cell apoptosis and by mixture of the cytokines IL-1β and IFN-γ, which induced a 2.3-fold increase in beta-cell apoptosis, compared to control conditions at 5.5 mM glucose (p<0.05, Fig. 2A,B). Addition of Nampt or NMN had no effect on beta-cell apoptosis in all conditions.

### Nampt and NMN Potentiate Glucose Stimulated Insulin Secretion in Human Islets

**Figure 3:**
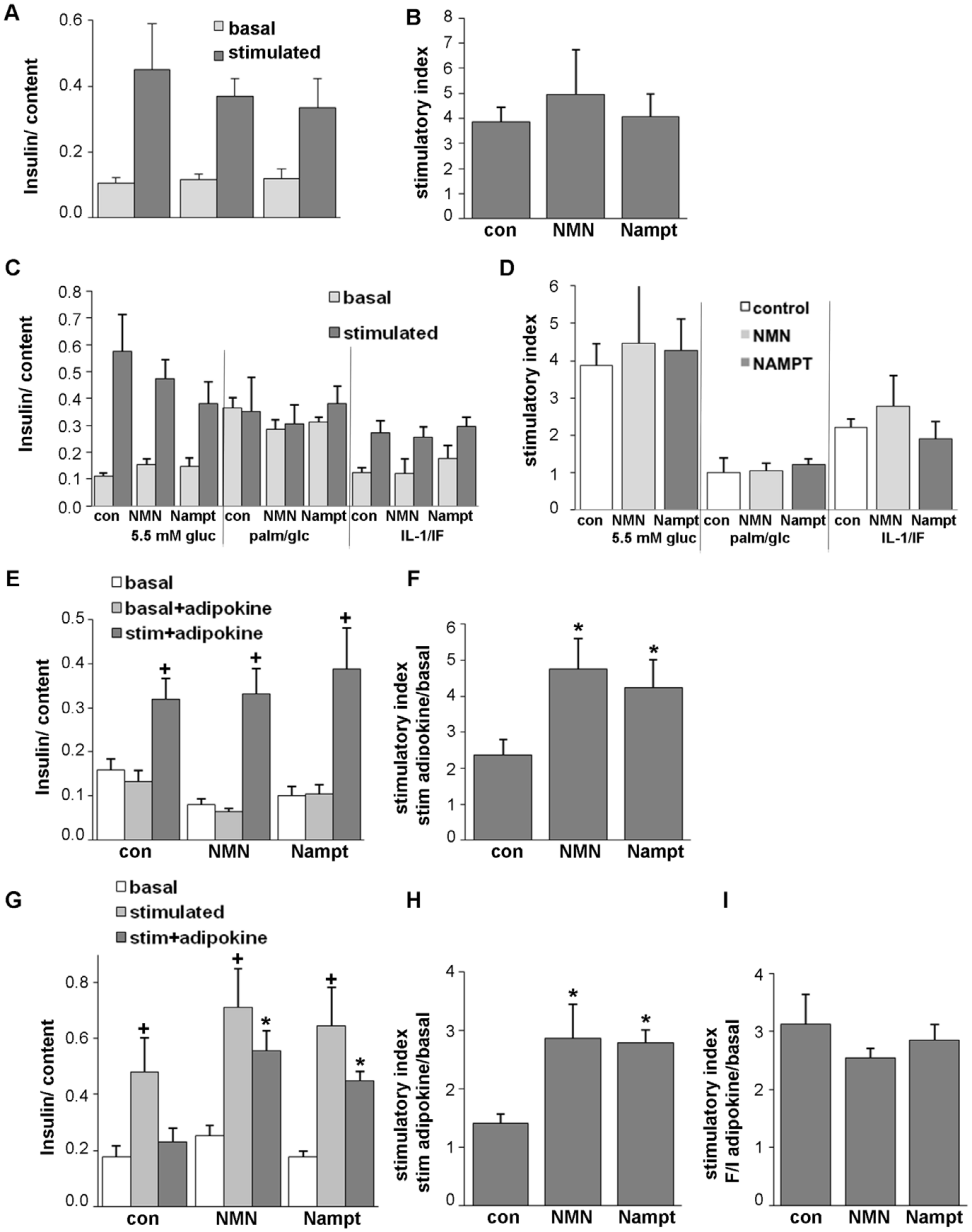
Nampt and NMN potentiate glucose stimulated insulin secretion (GSIS) in human islets. GSIS from human islets cultured on extracellular matrix coated dishes and chronically (**A–D**) or acutely (E–I) exposed to NMN (100 µM) and Nampt (2.5 ng/ml). (**A–D**) Islets were chronically exposed to the treatment conditions for 72 h (**A,B**: 5.5 mM glucose; **C,D**: 5.5 mM glucose, the mixture of 22.2 mM glucose/0.5 mM palmitate or 2 ng/ml IL-1β/1000 IU IFN-γ), medium was changed and GSIS performed in the absence of the treatment conditions. Basal and stimulated insulin secretion indicate the amount secreted during 1 h incubations at 2.8 (basal) and 16.7 mM (stimulated) glucose following the 72 h culture period and normalized to insulin content. The stimulatory index was calculated (**B,D**). (**E,F**) Islets were pre-cultured for 48h and then exposed to 2.8 mM glucose for 1 h (basal), to 2.8 mM glucose including the adipocytokines for 1 h (basal+adipokine) and another subsequent hour to 16.7 mM glucose including the adipocytokines (stim+adipokine). The stimulatory index was calculated (**F**). (**G, H**) Islets were pre-cultured for 48 h and then exposed to 2.8 mM glucose for 1 h (basal), to 16.7 mM glucose for 1 h (stimulated) and another subsequent hour to 16.7 mM glucose including the adipocytokines (stim+adipokine). The stimulatory index was calculated (**H**). (**I**) Stimulatory index from human islets exposed to 2.8 mM glucose (basal) and subsequently to 1 h exposure to IBMX (100 mM)/Forskolin (10 mM) with or without Nampt or NMN was calculated. Results are means ±SEM from triplicates from three independent experiments from three donors. *p<0.05 to the respective untreated control, ^+^p<0.05 to 2.8 mM basal glucose.

Since Nampt and NMN failed to protect human islets from apoptosis induced by diabetogenic conditions, we tested whether it may influence insulin secretion under basal conditions in culture. Human islets were chronically exposed to Nampt or NMN at 5.5 mM glucose for 72 h and GSIS was analysed thereafter. Nampt and its enzymatic product NMN did not influence beta-cell insulin secretion upon chronic exposure (Fig. 3A,B). Next, we investigated whether Nampt and NMN have an effect on long-term glucolipotoxicity and cytokine toxicity, induced by 72 h exposure of human islets to the mixture of 22.2 mM glucose and 0.5 mM palmitate or by the mixture of the cytokines IL-1β and IFN-γ. Glucose stimulated insulin secretion was determined at the end of the 72 h culture. Glucose/palmitate as well as the cytokine mixture severely reduced the stimulatory index (Fig. 3C,D; 3.8- and 1.8-fold respectively, p<0.05). Neither Nampt nor NMN changed GSIS in any of the conditions (Fig. 3C,D). This is in line with the above described lack of influence of Nampt and NMN on beta-cell survival (Fig. 2).

To determine the acute effect of Nampt and NMN on insulin secretion, we cultured the islets in the presence of the adipocytokine at low and high glucose concentrations for 1 h, respectively. At low glucose, Nampt and NMN elicited no significant effect on insulin secretion when compared to low glucose alone (Fig. 3E, basal +adipokine vs. basal). At high glucose conditions the GSIS was improved by Nampt and NMN. While glucose alone induced a 2.4-fold induction of insulin secretion, this induction was 2.0- and 1.8-fold induced by NMN and Nampt, respectively (p<0.05, Fig. 3E,F, stim+adipokine vs. basal), when compared to 16.7 mM glucose alone. To exclude exhaustive effects on beta-cell insulin secretion, which could have occurred after stimulation with high glucose concentrations, we repeated the experiment by testing adipocytokine effects only at high glucose conditions. Again, human islets were pre-cultured for 2 days at 5.5 mM glucose, basal glucose of 2.8 mM (Fig. 3G, basal) was added for 1 h followed by 1 h exposure to high glucose (16.7 mM) (Fig. 3G, stimulated) and then to high glucose in the presence of Nampt or NMN (Fig. 3G, stim+adipokine). All islets showed similar GSIS before the addition of the adipocytokine (Fig. 3G, stimulated). In contrast, islets which were stimulated a 2^nd^ subsequent hour with 16.7 mM glucose alone showed a decrease in GSIS (Fig. 3G, con, dark grey bar). Islets which were exposed to high glucose and Nampt or its enzymatic product NMN showed a restoration of insulin secretion, which was 2.0-fold increased by Nampt and NMN, respectively, when compared to 16.7 mM glucose alone (p<0.01, Fig. 3G,H, stim+adipokine vs. basal).

This effect of Nampt and NMN on the potentiation of glucose stimulated insulin secretion was dependent on the effect of glucose, since stimulation of insulin secretion with agents which raise intracellular levels of cAMP (forskolin and 3-isobutyl-Methylxanthine/IBMX) was not influenced by Nampt or NMN (Fig. 3I).

### NAMPT and NMN Increase NAD Level and Ameliorate NAD Depletion

**Figure 4:**
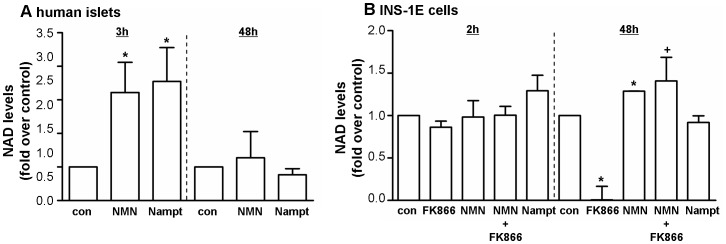
Nampt and NMN increase NAD level and ameliorate NAD depletion. Human islets (A) and INS-1E cells (B) were stimulated with NMN [100 µM] or Nampt [2.5 ng/ml] for a short time (2 or 3 h) or a long time exposure (48 h). NAD level were normalised to the total protein amount in each sample. FK866 [10 nM], a specific Nampt inhibitor, was used as a positive control in INS-1E cells. * p<0.05 compared to con (serum free medium);+p<0.05 compared to FK866 treated cells.

NAD level increased in human islets after short time incubation with NMN (2.2-fold) or Nampt (2.6-fold). Incubation for a longer time did not change NAD level in human islets (Fig. 4A). In INS-1E cells short time incubation with NMN or Nampt did not alter intracellular NAD level whereas the NAD level after 48 h incubation were slightly increased by NMN (0.3-fold). After 48 h, NMN restored intracellular NAD level after NAD depletion caused by FK866, a specific Nampt inhibitor (Fig. 4B).

## Discussion

Because of the controversial data concerning the effects of adiponectin, leptin and Nampt on beta-cell survival [9]; [11]; [22]–[25], we aimed to evaluate whether or not these adipocytokines affect beta-cell viability, cytotoxicity and apoptosis. For the first time we also show presence of Nampt in beta-cells and human islets (Fig. S4A,B) and effects of Nampt and its enzyme product NMN on human beta-cell function and survival.

Previously, adiponectin (gAcrp30) and leptin were found to strongly inhibit palmitate-induced apoptosis, with a weaker effect on cytokine-induced apoptosis [9], [11]. In contrast, another previous study showed that leptin and high glucose levels induce apoptosis in human and rat beta-cells [11]. However, we did not find any changes of INS-1E cell viability, cytotoxicity or apoptosis after stimulation with the adipocytokines. Neither NF-κB-, caspase-3- nor p53- mediated apoptotic pathways were influenced by adiponectin, leptin or Nampt under the experimental conditions we used. In cell line experiments, a number of experimental factors could explain such discrepancies; differences between cell lines (Min6 or INS-1E cells), cell passages or starvation conditions could result in different experimental outcomes. In our study, beta-cells were starved before the experiments started, whereas in a previous study, non-starving conditions were used [9]. The leptin and adiponectin concentrations in our study are in agreement with other *in vitro* studies [9], [11], [32]. Leptin at 200 ng/ml is within the upper range that is measured in obese people [33], [34]. Adiponectin concentrations range from 1–5000 ng/ml in different studies [35], [36] and strongly correlate with insulin sensitivity and beta-cell compensation [37]. In line with our results, also Staiger *et al.* did not detect any effects of adiponectin on beta-cell survival or insulin secretion despite functionally active receptors in human islets [20].

Results from clinical studies show that visfatin/Nampt levels are elevated in nonobese and obese patients with T2DM compared with control subjects [26]. Additionally, circulating serum levels of Nampt are elevated in obese compared to lean children [38], suggesting that Nampt is associated with beta-cell function in humans. To elucidate whether the demonstrated effects of Nampt depend upon its enzymatic activity, we tested NMN, the enzyme product of Nampt. In our study, physiological adipocytokine concentrations were used to test their effects. Nampt serum levels are 2.22 ng/ml in healthy adults and increase up to 2.75 ng/ml in patients with T2DM [39], thus 2.5 ng/ml was used in our cell culture studies. This amount corresponds to approximately 0.023 nM for the Nampt dimer, which is the molecular form of Nampt in human serum that is enzymatically active [40]. No physiological human serum concentrations for NMN have been published so far. Data from mice report a plasma concentration of 80–90 µM [21], thus, a dose of 100 µM NMN was used in our study. Nampt has also been shown to exert enzyme-independent cytokine-like anti-apoptotic effects [24], [41]. There were no protective effects of Nampt and NMN on the beta-cell line INS-1E and human islets using multiple tested assays. Nampt and NMN also had no effect on the function of human islets upon chronic exposure. Further, pro-apoptotic signaling pathways, such as activation of p-53 and NF-κB were activated by cytokines in our study, which is in line with numerous previous publications [7], [42], [43], and were not modified by Nampt, although other adipocytokines can modify such pathways [9], [11]. In Min6 beta-cells, palmitate-induced beta-cell apoptosis was inhibited by Nampt [23]. However, in that study, a Nampt concentration of 100 nM was tested, which is approximately 4300-fold higher than in physiological conditions.

In our study, Nampt as well as NMN did not change basal insulin release, but acutely potentiated GSIS under high glucose conditions, which is reminiscent of the effects of Glucagon-like peptide-1 (GLP-1), being only affective at high glucose concentrations, but additionally, GLP-1 shows protective effects on beta-cell survival [44]. Nampt-mediated systemic NAD biosynthesis is critical for beta-cell function and for the regulation of glucose homeostasis [21]. Nampt heterozygous (Nampt^+/−^) female mice show impaired glucose tolerance due to a defect in GSIS. The administration of NMN has been demonstrated to restore GSIS in Nampt^(+/−)^ mice *in vivo* and in islets *in vitro* [21] and also to protect against cytokine-mediated impairment of beta-cell function in mouse islets [22]. This strongly indicates that the observed defects are due to a lack of Nampt-mediated NAD biosynthesis. According to this, our data revealed that NMN restores intracellular NAD level after depletion caused by FK866, a specific Nampt inhibitor. As an NAD biosynthetic enzyme, Nampt regulates the activity of NAD-consuming enzymes such as sirtuins, which are involved in cellular homeostasis, glucose metabolism and stress responses [45]. We measured increased intracellular NAD level after short time incubation with NMN and Nampt in human islets which might explain the beneficial effects on GSIS. In a previous study of Bordone *et al*. sirtuin 1 (Sirt1) promoted insulin secretion in pancreatic beta-cells in response to glucose partly through repression of uncoupling protein 2 (Ucp2) and consequently increased levels of ATP [46]. We could not found any changes in ATP level (Fig. S4C) after stimulation with Nampt and NMN for 2, 48 and 72 h. Probably, the changes in NAD level are too small to detect alterations in ATP concentrations. Further, pancreatic beta-cell-specific Sirt1-overexpressing (BESTO) transgenic mice exhibited enhanced GSIS and improved glucose tolerance [47]. Additionally, it was found that old BESTO mice have significantly reduced plasma NMN levels and lost their ability to GSIS. NMN administration restored the improved glucose tolerance and enhanced GSIS in these aged female BESTO mice [48].

Our findings indicate that Nampt and NMN did not influence beta-cell survival. However, targeting NAD biosynthesis might represent novel therapeutic strategies in the control of beta-cell function.

## Supporting Information

Figure S1 **Expression of the adiponectin receptors (AdipoR1, AdipoR2) and the leptin receptor.** Demonstration of mRNA expression of the adiponectin receptors (AdipoR1, AdipoR2) and the leptin receptor (LepR/Ob-R) in INS-1E cells and in visceral fat taken from rats.
(TIF)

Figure S2 **Oleate protects from palmitate induced apoptosis in INS-1E cells.** INS-1E cells were exposed to palmitate and oleate at increasing concentrations alone (**A**) or in combination for 72 h (**B**) or 24 h (**C**). Viability was measured by WST-1 analysis (**A,B**) and cytotoxicity was analyzed by measuring the release of adenylate kinase in the supernatant (**C**). Data show the mean ± SEM of quadruplicates of three independent experiments. *p<0.05 to untreated control, **p<0.05 to palmitate treated cells. (**D**) Western blot analysis was performed for control cells, 0.5 mM palmitate (pal) treated cells, 0.5 mM oleate (ol) treated cells and for the combination (pal+ol) for full length and cleaved caspase-3. GAPDH was used as loading control. All panels show one typical blot out of three independent experiments.
(TIF)

Figure S3 **Cytokines increased apoptosis in INS-1E cells.** INS-1E cells were exposed to the cytokine combination (10+10 ng/ml IL/IF) and the adipocytokines (200 ng/ml leptin, 167 ng/ml adiponectin and 2.5 ng/ml Nampt) for 48 h. Apoptosis in INS-1E cells was assessed by FITC Annexin V (An) and propidium iodide (PI) staining and flow cytometric analysis. For each sample, 10,000 cells were counted. An-positive and double-stained An/PI positive cells were defined as apoptotic cells.
(TIF)

Figure S4 **Nampt is expressed in human islets and the beta-cell line INS-1E. (A)** Nampt mRNA was analysed in human islets and INS-1E cells by PCR. Human Nampt was amplified using the primers: forward ATGAATCCTGCGGCAGAAGC and reverse CTAATGATGTGCTGCTTCCAGT [40]. To detect Nampt mRNA in rats forward primer CCACCGACTCGTACAAGGTT and reverse primer ACTTCTTTGGCCTCCTGGAT were used. **(B)** Nampt protein was detected in lysates [10 µg protein] by using a monoclonal antibody (1∶5000) in 5% non-fat dry milk (OMNI379, Axxora, Lörrach, Germany) in human islets and INS-1E cells. For normalisation GAPDH was used. **(C)** ATP level were measured according to manufacturer’s instructions (CellTiter-Glo® Luminescent Cell Viability Assay, Promega, Madison, WI, USA) after 2, 48 and 72 h with NMN [100 µM], Nampt [2,5 ng/ml] or FK866 [10 nM], a specific Nampt inhibitor.
(TIF)

## References

[1] Wang C, Guan Y, Yang J (2010) Cytokines in the Progression of Pancreatic beta-Cell Dysfunction. Int J Endocrinol 2010: 515136. 21113299 10.1155/2010/515136 PMC2989452

[2] Stumvoll M, Goldstein BJ, van Haeften TW (2005) Type 2 diabetes: principles of pathogenesis and therapy. Lancet 365: 1333–1346. 15823385 10.1016/S0140-6736(05)61032-X

[3] Pukel C, Baquerizo H, Rabinovitch A (1988) Destruction of rat islet cell monolayers by cytokines. Synergistic interactions of interferon-gamma, tumor necrosis factor, lymphotoxin, and interleukin 1. Diabetes 37: 133–136. 3121415 10.2337/diab.37.1.133

[4] Maedler K, Dharmadhikari G, Schumann DM, Storling J (2009) Interleukin-1 beta targeted therapy for type 2 diabetes. Expert Opin Biol Ther 9: 1177–1188. 19604125 10.1517/14712590903136688

[5] Eizirik DL, Mandrup-Poulsen T (2001) A choice of death–the signal-transduction of immune-mediated beta-cell apoptosis. Diabetologia 44: 2115–2133. 11793013 10.1007/s001250100021

[6] Poitout V, Robertson RP (2008) Glucolipotoxicity: fuel excess and beta-cell dysfunction. Endocr Rev 29: 351–366. 18048763 10.1210/er.2007-0023 PMC2528858

[7] Kharroubi I, Ladriere L, Cardozo AK, Dogusan Z, Cnop M, et al (2004) Free fatty acids and cytokines induce pancreatic beta-cell apoptosis by different mechanisms: role of nuclear factor-kappaB and endoplasmic reticulum stress. Endocrinology 145: 5087–5096. 15297438 10.1210/en.2004-0478

[8] Maedler K, Spinas GA, Dyntar D, Moritz W, Kaiser N, et al (2001) Distinct effects of saturated and monounsaturated fatty acids on beta-cell turnover and function. Diabetes 50: 69–76. 11147797 10.2337/diabetes.50.1.69

[9] Rakatzi I, Mueller H, Ritzeler O, Tennagels N, Eckel J (2004) Adiponectin counteracts cytokine- and fatty acid-induced apoptosis in the pancreatic beta-cell line INS-1. Diabetologia 47: 249–258. 14722646 10.1007/s00125-003-1293-3

[10] Cunha DA, Hekerman P, Ladriere L, Bazarra-Castro A, Ortis F, et al (2008) Initiation and execution of lipotoxic ER stress in pancreatic beta-cells. J Cell Sci 121: 2308–2318. 18559892 10.1242/jcs.026062 PMC3675788

[11] Maedler K, Schulthess FT, Bielman C, Berney T, Bonny C, et al (2008) Glucose and leptin induce apoptosis in human beta-cells and impair glucose-stimulated insulin secretion through activation of c-Jun N-terminal kinases. FASEB J 22: 1905–1913. 18263705 10.1096/fj.07-101824

[12] Seufert J (2004) Leptin effects on pancreatic beta-cell gene expression and function. Diabetes 53 Suppl 1S152–S158. 14749281 10.2337/diabetes.53.2007.s152

[13] Kieffer TJ, Heller RS, Habener JF (1996) Leptin receptors expressed on pancreatic beta-cells. Biochem Biophys Res Commun 224: 522–527. 8702421 10.1006/bbrc.1996.1059

[14] Maedler K, Sergeev P, Ehses JA, Mathe Z, Bosco D, et al (2004) Leptin modulates beta cell expression of IL-1 receptor antagonist and release of IL-1beta in human islets. Proc Natl Acad Sci U S A 101: 8138–8143. 15141093 10.1073/pnas.0305683101 PMC419570

[15] Koerner A, Kratzsch J, Kiess W (2005) Adipocytokines: leptin–the classical, resistin–the controversical, adiponectin–the promising, and more to come. Best Pract Res Clin Endocrinol Metab 19: 525–546. 16311215 10.1016/j.beem.2005.07.008

[16] Kharroubi I, Rasschaert J, Eizirik DL, Cnop M (2003) Expression of adiponectin receptors in pancreatic beta cells. Biochem Biophys Res Commun 312: 1118–1122. 14651988 10.1016/j.bbrc.2003.11.042

[17] Kadowaki T, Yamauchi T (2005) Adiponectin and adiponectin receptors. Endocr Rev 26: 439–451. 15897298 10.1210/er.2005-0005

[18] Gu W, Li X, Liu C, Yang J, Ye L, et al (2006) Globular adiponectin augments insulin secretion from pancreatic islet beta cells at high glucose concentrations. Endocrine 30: 217–221. 17322583 10.1385/ENDO:30:2:217

[19] Winzell MS, Nogueiras R, Dieguez C, Ahren B (2004) Dual action of adiponectin on insulin secretion in insulin-resistant mice. Biochem Biophys Res Commun 321: 154–160. 15358228 10.1016/j.bbrc.2004.06.130

[20] Staiger K, Stefan N, Staiger H, Brendel MD, Brandhorst D, et al (2005) Adiponectin is functionally active in human islets but does not affect insulin secretory function or beta-cell lipoapoptosis. J Clin Endocrinol Metab 90: 6707–6713. 16204361 10.1210/jc.2005-0467

[21] Revollo JR, Korner A, Mills KF, Satoh A, Wang T, et al (2007) Nampt/PBEF/Visfatin regulates insulin secretion in beta cells as a systemic NAD biosynthetic enzyme. Cell Metab 6: 363–375. 17983582 10.1016/j.cmet.2007.09.003 PMC2098698

[22] Caton PW, Kieswich J, Yaqoob MM, Holness MJ, Sugden MC (2011) Nicotinamide mononucleotide protects against pro-inflammatory cytokine-mediated impairment of mouse islet function. Diabetologia. 10.1007/s00125-011-2288-0 21901281

[23] Cheng Q, Dong WP, Qian L, Wu JC, Peng YD (2011) Visfatin inhibits apoptosis of pancreatic {beta}-cell line, MIN6, via the mitogen-activated protein kinase/phosphoinositide 3-kinase pathway. J Mol Endocrinol. 10.1530/JME-10-0106 21471274

[24] Li Y, Zhang Y, Dorweiler B, Cui D, Wang T, et al (2008) Extracellular Nampt promotes macrophage survival via a nonenzymatic interleukin-6/STAT3 signaling mechanism. J Biol Chem 283: 34833–34843. 18945671 10.1074/jbc.M805866200 PMC2596403

[25] Romacho T, Azcutia V, Vazquez-Bella M, Matesanz N, Cercas E, et al (2009) Extracellular PBEF/NAMPT/visfatin activates pro-inflammatory signalling in human vascular smooth muscle cells through nicotinamide phosphoribosyltransferase activity. Diabetologia 52: 2455–2463. 19727662 10.1007/s00125-009-1509-2

[26] El Mesallamy HO, Kassem DH, El Demerdash E, Amin AI (2011) Vaspin and visfatin/Nampt are interesting interrelated adipokines playing a role in the pathogenesis of type 2 diabetes mellitus. Metabolism 60: 63–70. 20605615 10.1016/j.metabol.2010.04.008

[27] Merglen A, Theander S, Rubi B, Chaffard G, Wollheim CB, et al (2004) Glucose sensitivity and metabolism-secretion coupling studied during two-year continuous culture in INS-1E insulinoma cells. Endocrinology 145: 667–678. 14592952 10.1210/en.2003-1099

[28] Maedler K, Oberholzer J, Bucher P, Spinas GA, Donath MY (2003) Monounsaturated fatty acids prevent the deleterious effects of palmitate and high glucose on human pancreatic beta-cell turnover and function. Diabetes 52: 726–733. 12606514 10.2337/diabetes.52.3.726

[29] Sauter NS, Schulthess FT, Galasso R, Castellani LW, Maedler K (2008) The antiinflammatory cytokine interleukin-1 receptor antagonist protects from high-fat diet-induced hyperglycemia. Endocrinology 149: 2208–2218. 18239070 10.1210/en.2007-1059 PMC2734491

[30] Shu L, Matveyenko AV, Kerr-Conte J, Cho JH, McIntosh CH, et al (2009) Decreased TCF7L2 protein levels in type 2 diabetes mellitus correlate with downregulation of GIP- and GLP-1 receptors and impaired beta-cell function. Hum Mol Genet 18: 2388–2399. 19386626 10.1093/hmg/ddp178 PMC2722186

[31] Eitel K, Staiger H, Brendel MD, Brandhorst D, Bretzel RG, et al (2002) Different role of saturated and unsaturated fatty acids in beta-cell apoptosis. Biochem Biophys Res Commun 299: 853–856. 12470657 10.1016/s0006-291x(02)02752-3

[32] Kulkarni RN, Wang ZL, Wang RM, Hurley JD, Smith DM, et al (1997) Leptin rapidly suppresses insulin release from insulinoma cells, rat and human islets and, in vivo, in mice. J Clin Invest 100: 2729–2736. 9389736 10.1172/JCI119818 PMC508476

[33] De Marinis L, Bianchi A, Mancini A, Gentilella R, Perrelli M, et al (2004) Growth hormone secretion and leptin in morbid obesity before and after biliopancreatic diversion: relationships with insulin and body composition. J Clin Endocrinol Metab 89: 174–180. 14715846 10.1210/jc.2002-021308

[34] Chan JL, Heist K, DePaoli AM, Veldhuis JD, Mantzoros CS (2003) The role of falling leptin levels in the neuroendocrine and metabolic adaptation to short-term starvation in healthy men. J Clin Invest 111: 1409–1421. 12727933 10.1172/JCI17490 PMC154448

[35] Coppola A, Marfella R, Coppola L, Tagliamonte E, Fontana D, et al (2009) Effect of weight loss on coronary circulation and adiponectin levels in obese women. Int J Cardiol 134: 414–416. 18378021 10.1016/j.ijcard.2007.12.087

[36] Xiang AH, Kawakubo M, Trigo E, Kjos SL, Buchanan TA (2010) Declining beta-cell compensation for insulin resistance in Hispanic women with recent gestational diabetes mellitus: association with changes in weight, adiponectin, and C-reactive protein. Diabetes Care 33: 396–401. 19933993 10.2337/dc09-1493 PMC2809290

[37] Yamauchi T, Kamon J, Waki H, Terauchi Y, Kubota N, et al (2001) The fat-derived hormone adiponectin reverses insulin resistance associated with both lipoatrophy and obesity. Nat Med 7: 941–946. 11479627 10.1038/90984

[38] Friebe D, Neef M, Kratzsch J, Erbs S, Dittrich K, et al (2011) Leucocytes are a major source of circulating nicotinamide phosphoribosyltransferase (NAMPT)/pre-B cell colony (PBEF)/visfatin linking obesity and inflammation in humans. Diabetologia 54: 1200–1211. 21298414 10.1007/s00125-010-2042-z PMC3071946

[39] Retnakaran R, Youn BS, Liu Y, Hanley AJ, Lee NS, et al (2008) Correlation of circulating full-length visfatin (PBEF/NAMPT) with metabolic parameters in subjects with and without diabetes: a cross-sectional study. Clin Endocrinol (Oxf) 69: 885–893. 18410550 10.1111/j.1365-2265.2008.03264.x

[40] Korner A, Garten A, Bluher M, Tauscher R, Kratzsch J, et al (2007) Molecular characteristics of serum visfatin and differential detection by immunoassays. J Clin Endocrinol Metab 92: 4783–4791. 17878256 10.1210/jc.2007-1304

[41] Dahl TB, Haukeland JW, Yndestad A, Ranheim T, Gladhaug IP, et al (2010) Intracellular nicotinamide phosphoribosyltransferase protects against hepatocyte apoptosis and is down-regulated in nonalcoholic fatty liver disease. J Clin Endocrinol Metab 95: 3039–3047. 20392873 10.1210/jc.2009-2148

[42] Lee BW, Chun SW, Kim SH, Lee Y, Kang ES, et al (2011) Lithospermic acid B protects beta-cells from cytokine-induced apoptosis by alleviating apoptotic pathways and activating anti-apoptotic pathways of Nrf2-HO-1 and Sirt1. Toxicol Appl Pharmacol 252: 47–54. 21295052 10.1016/j.taap.2011.01.018

[43] Sarkar SA, Kutlu B, Velmurugan K, Kizaka-Kondoh S, Lee CE, et al (2009) Cytokine-mediated induction of anti-apoptotic genes that are linked to nuclear factor kappa-B (NF-kappaB) signalling in human islets and in a mouse beta cell line. Diabetologia 52: 1092–1101. 19343319 10.1007/s00125-009-1331-x

[44] Drucker DJ (2003) Glucagon-like peptide-1 and the islet beta-cell: augmentation of cell proliferation and inhibition of apoptosis. Endocrinology 144: 5145–5148. 14645210 10.1210/en.2003-1147

[45] Imai S (2011) Dissecting systemic control of metabolism and aging in the NAD World: the importance of SIRT1 and NAMPT-mediated NAD biosynthesis. FEBS Lett 585: 1657–1662. 21550345 10.1016/j.febslet.2011.04.060 PMC3104082

[46] Bordone L, Motta MC, Picard F, Robinson A, Jhala US, et al (2006) Sirt1 regulates insulin secretion by repressing UCP2 in pancreatic beta cells. PLoS Biol 4: e31. 16366736 10.1371/journal.pbio.0040031 PMC1318478

[47] Moynihan KA, Grimm AA, Plueger MM, Bernal-Mizrachi E, Ford E, et al (2005) Increased dosage of mammalian Sir2 in pancreatic beta cells enhances glucose-stimulated insulin secretion in mice. Cell Metab 2: 105–117. 16098828 10.1016/j.cmet.2005.07.001

[48] Ramsey KM, Mills KF, Satoh A, Imai S (2008) Age-associated loss of Sirt1-mediated enhancement of glucose-stimulated insulin secretion in beta cell-specific Sirt1-overexpressing (BESTO) mice. Aging Cell 7: 78–88. 18005249 10.1111/j.1474-9726.2007.00355.x PMC2238677

