# Correction: The Adipocytokine Nampt and Its Product NMN Have No Effect on Beta-Cell Survival but Potentiate Glucose Stimulated Insulin Secretion

**DOI:** 10.1371/journal.pone.0270243

**Published:** 2022-06-16

**Authors:** The PLOS ONE Editors

After publication of this article [1], concerns were raised about vertical discontinuities in the western blots in Fig 1G.

**Fig 1:**
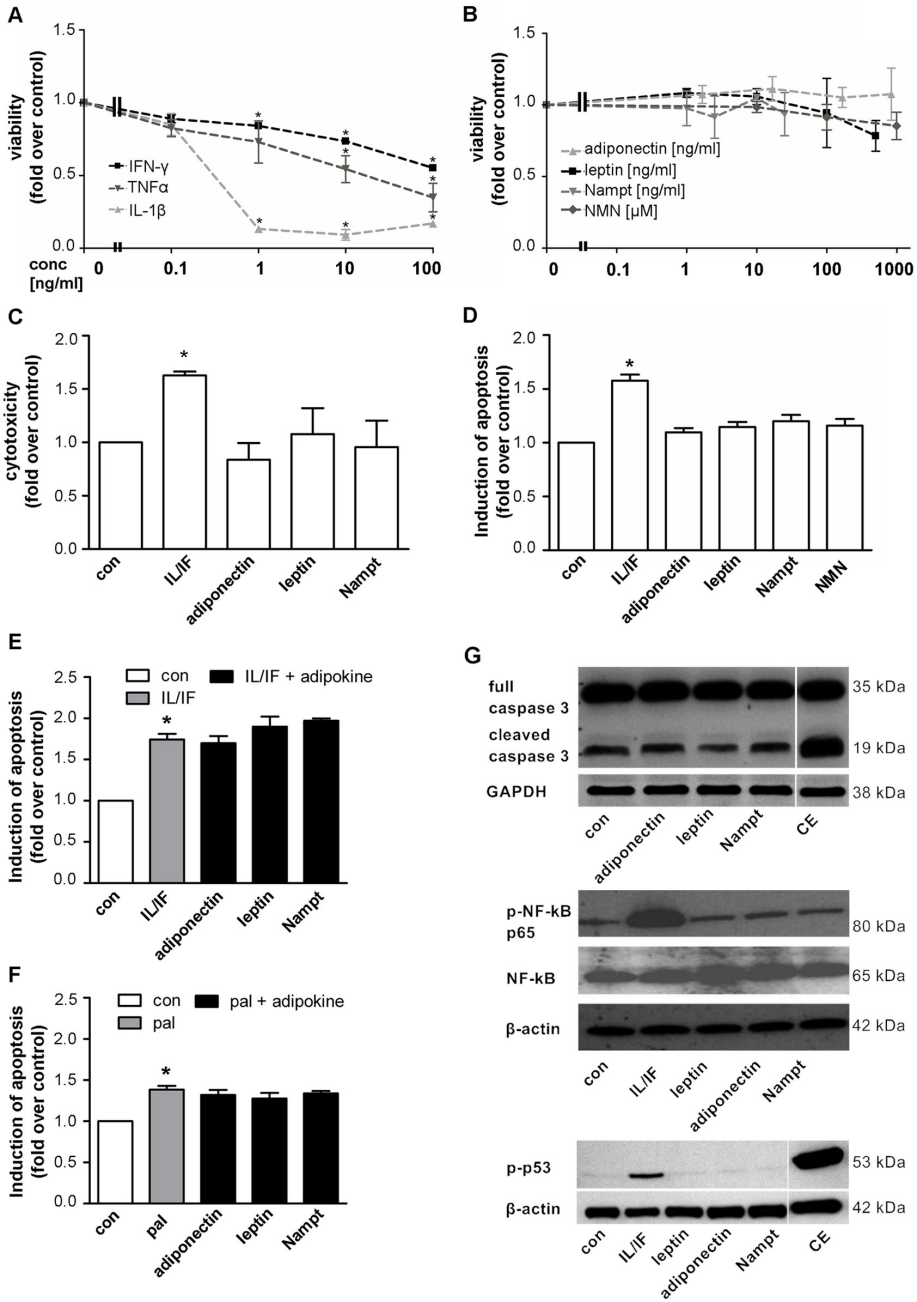
The adipocytokines leptin, adiponectin, Nampt and NMN have no direct effects on beta-cell survival in INS-1E cells. INS-1E cells were kept under serum-free conditions 24 h before and during the 48 h experiment. (**A,B**) INS-1E cells were exposed to cytokines (**A**: IL-1β, IFN-γ or TNFα) or adipocytokines (**B**: adiponectin, leptin, Nampt, NMN) at the indicated concentrations for 48 h and cell viability was measured by WST-1 assay. Data are shown as means ±SEM of 3 independent experiments performed in triplicates. Statistical analyses were performed by one-way ANOVA with Bonferroni’s Multiple Comparison Test as posthoc test. **C,D**: INS-1E cells were exposed to adipocytokines (adiponectin 167 ng/ml, leptin 200 ng/ml, Nampt 2.5 ng/ml, NMN 100 μM) or a cytokine combination (10 ng/ml IL-1β+10 ng/ml IFN-γ) for 48 h. Cytotoxicity (**C**) was analyzed by measuring the release of adenylate kinase into the supernatant and (**D**) apoptosis was measured by FITC-annexinV (An) and propidium iodide (PI) staining and subsequent flow cytometric analysis of An-positive and double An/PI positive cells. Results were expressed relative to cells exposed to serum free medium (con) and as means ±SEM of three independent experiments performed in triplicates. **E,F**: INS-1E cells were exposed to a cytokine combination (IL/IF; 10 ng/ml IL-1β+10 ng/ml IFN-γ) (**E**) or 0.25 mM palmitate (pal) (**F**) for 48 h in the absence or presence of the adipocytokines (167 ng/ml adiponectin, 200 ng/ml leptin, 2.5 ng/ml Nampt) and induction of apoptosis was measured by An/PI staining and flow cytometric analysis. Data are shown as means ±SEM of triplicates of three independent experiments. Statistical analyses were performed by students t-test. **G**: INS-1E cells were exposed to the adipocytokines adiponectin (167 ng/ml), leptin (200 ng/ml) or Nampt (2.5 ng/ml) or a combination of camptothecin (2 μM) and etoposide (85 μM; CE, **upper and lower panel**) or a cytokine combination (10 ng/ml IL-1β+10 ng/ml IFN-γ, **middle and lower panel**). Western blot analyses were performed for full length and cleaved caspase-3 (**upper panel**), phospho-NF-κB p65 (Ser536) and NF-κB p65 (**middle panel**) and phospho-p53 (Ser15) (**lower panel**). GAPDH or beta-actin were used as loading control. All panels show one typical blot out of three independent experiments. *p<0.05 compared to untreated control.

The *PLOS ONE* Editors followed up with the authors, who have provided the following additional information:

The last author stated that a single lane has been spliced from all blots of the upper and lower panels of Fig 1G as this lane is not part of the data reported in article [1]. The authors noted that lanes 1–5 and lane 6 in the bottom two blots in Fig 1G (p-p53 and β-actin) were spliced from their original position in the raw blots, in order to display them together. In addition, lanes 1–4 and lane 5 in the top three blots in Fig 1G (full caspase 3, cleaved caspase 3 and GAPDH) were spliced from their original position in the raw blots, in order to display them together. The corresponding author stated that the lanes are non-contiguous but from the same gels. Here the authors provide a revised version of Fig 1G in which the splice lines are denoted by vertical white lines. Underlying data from the original experiment reported in Fig 1G are in S1 File.

Raw data underlying the remaining results reported in the article are available from the corresponding author.

## Supporting information

S1 File Additional information for Fig 1G. (PDF)

## References

[1] Spinnler R, Gorski T, Stolz K, Schuster S, Garten A, Beck-Sickinger AG, et al. (2013) The Adipocytokine Nampt and Its Product NMN Have No Effect on Beta-Cell Survival but Potentiate Glucose Stimulated Insulin Secretion. PLoS ONE 8(1): e54106. doi: 10.1371/journal.pone.0054106 23342086 PMC3546920

