# Supplementary figures and images for: The Adipocytokine Nampt and Its Product NMN Have No Effect on Beta-Cell Survival but Potentiate Glucose Stimulated Insulin Secretion
Source: PLoS One. 2013 Jan 16;8(1):e54106. doi: 10.1371/journal.pone.0054106 (PMC3546920; doi:10.1371/journal.pone.0054106)

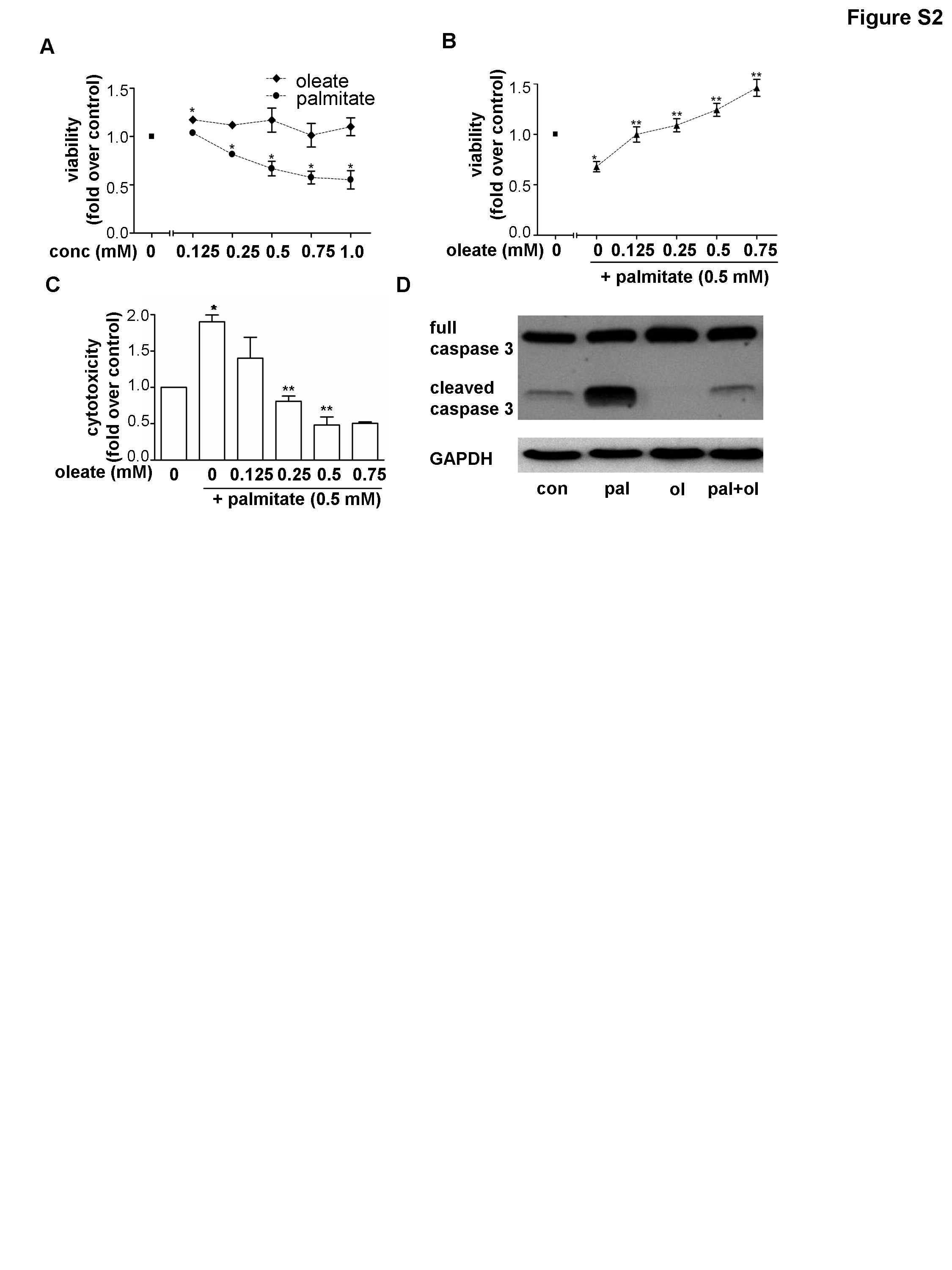

Supplement: Figure S2 — Oleate protects from palmitate induced apoptosis in INS-1E cells. INS-1E cells were exposed to palmitate and oleate at increasing concentrations alone (A) or in combination for 72 h (B) or 24 h (C). Viability was measured by WST-1 analysis (A,B) and cytotoxicity was analyzed by measuring the release of adenylate kinase in the supernatant (C). Data show the mean ± SEM of quadruplicates of three independent experiments. *p<0.05 to untreated control, **p<0.05 to palmitate treated cells. (D) Western blot analysis was performed for control cells, 0.5 mM palmitate (pal) treated cells, 0.5 mM oleate (ol) treated cells and for the combination (pal+ol) for full length and cleaved caspase-3. GAPDH was used as loading control. All panels show one typical blot out of three independent experiments. (TIF) [file pone.0054106.s002.tif]

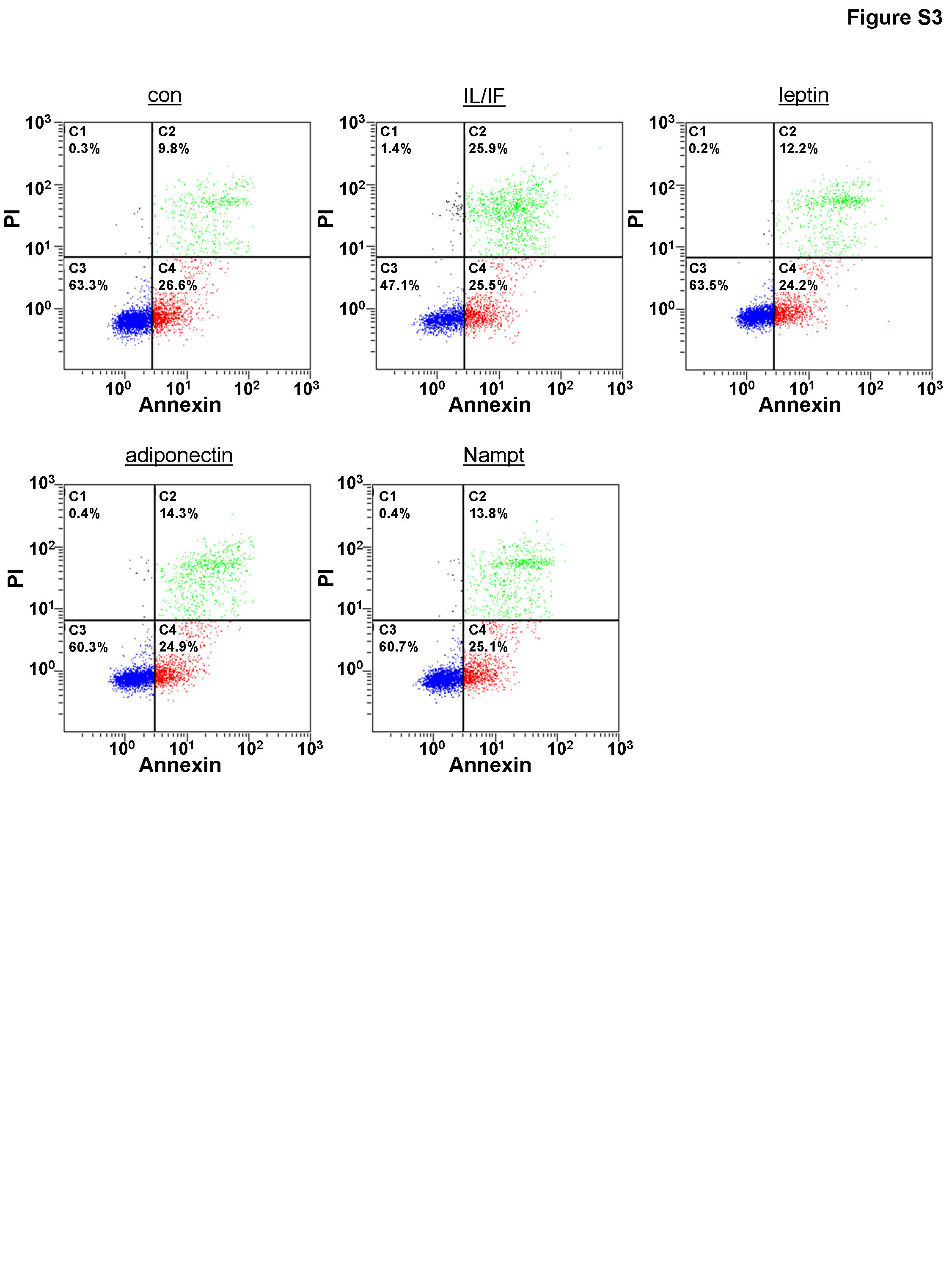

Supplement: Figure S3 — Cytokines increased apoptosis in INS-1E cells. INS-1E cells were exposed to the cytokine combination (10+10 ng/ml IL/IF) and the adipocytokines (200 ng/ml leptin, 167 ng/ml adiponectin and 2.5 ng/ml Nampt) for 48 h. Apoptosis in INS-1E cells was assessed by FITC Annexin V (An) and propidium iodide (PI) staining and flow cytometric analysis. For each sample, 10,000 cells were counted. An-positive and double-stained An/PI positive cells were defined as apoptotic cells. (TIF) [file pone.0054106.s003.tif]
