# Supplementary material for: Correction: The Adipocytokine Nampt and Its Product NMN Have No Effect on Beta-Cell Survival but Potentiate Glucose Stimulated Insulin Secretion
Source: PLoS One. 2022 Jun 16;17(6):e0270243. doi: 10.1371/journal.pone.0270243 (PMC9202939; doi:10.1371/journal.pone.0270243)
Supplement: S1 File — (PDF) [file pone.0270243.s001.pdf]

Uncut raw blots Fig.1G: PLoS One 2013;8(1):e54106

Phospho-p53 blot/Normalisierung beta-Aktin

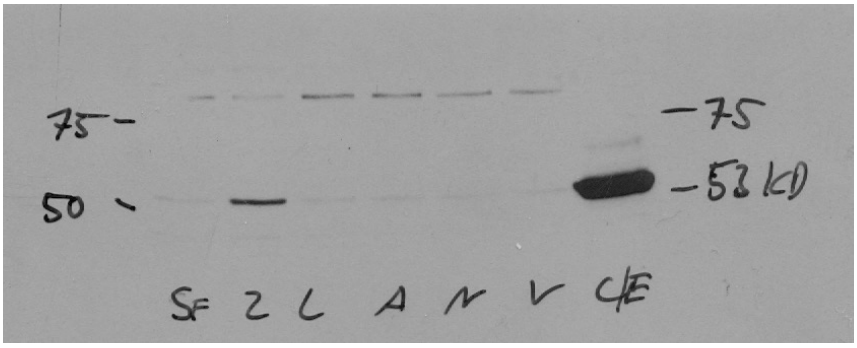

Phospho-p53

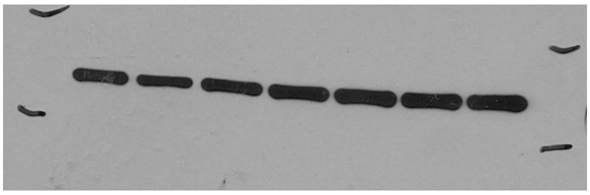

Beta-aktin

1 2 3 4 5 6 7

1: serum free control
2: cytokines (IL1beta/IFNgamma)
3: Leptin
4: Adiponectin
5: Nampt
6: other treatment (unrelated to published data)
7: Camptothecin/Etoposid (+control)

Uncut raw blots Fig.1G: PLoS One 2013;8(1):e54106

Caspase-3/cleaved caspase-3 blot/ GAPDH

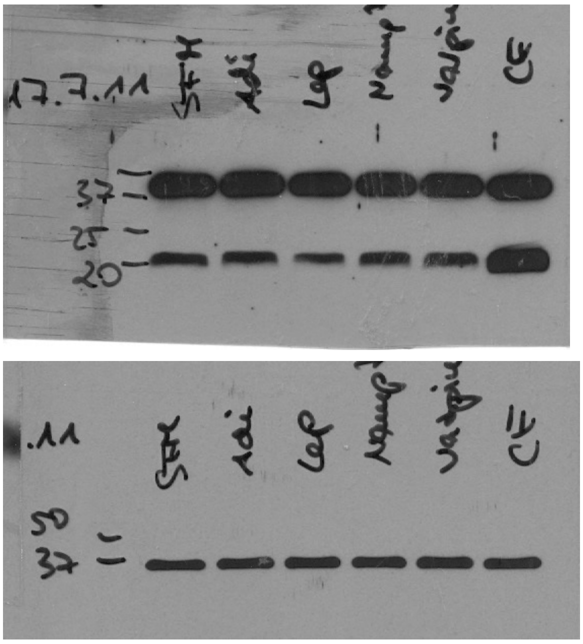

Caspase 3

Cleaved caspase 3

GAPDH

1 2 3 4 5 6

1: serum free control
2: Adiponectin
3: Leptin
4: Nampt
5: other treatment (unrelated to published data)
6: Camptothecin/Etoposid (+control)
